# Supplementary material for: Host-derived Interleukin 1α induces an immunosuppressive tumor microenvironment via regulating monocyte-to-macrophage differentiation
Source: bioRxiv. 2024 May 5:2024.05.03.592354. Preprint. [Version 1] doi: 10.1101/2024.05.03.592354 (PMC11092773; doi:10.1101/2024.05.03.592354)
Supplement: Supplement 1 [file NIHPP2024.05.03.592354v1-supplement-1.pdf]

bioRxiv preprint doi: https://doi.org/10.1101/2024.05.03.592354; this version posted May 5, 2024. The copyright holder for this preprint (which was not certified by peer review) is the author/funder, who has granted bioRxiv a license to display the preprint in perpetuity. It is made available under aCC-BY 4.0 International license.

Supplementary Materials for

Host-derived Interleukin 1α induces an immunosuppressive tumor microenvironment via regulating monocyte-to-macrophage differentiation

Manikanda Raja Keerthi Raja et al.

*Corresponding author. Email: hchen@biol.sc.edu

bioRxiv preprint doi: https://doi.org/10.1101/2024.05.03.592354; this version posted May 5, 2024. The copyright holder for this preprint (which was not certified by peer review) is the author/funder, who has granted bioRxiv a license to display the preprint in perpetuity. It is made available under aCC-BY 4.0 International license.

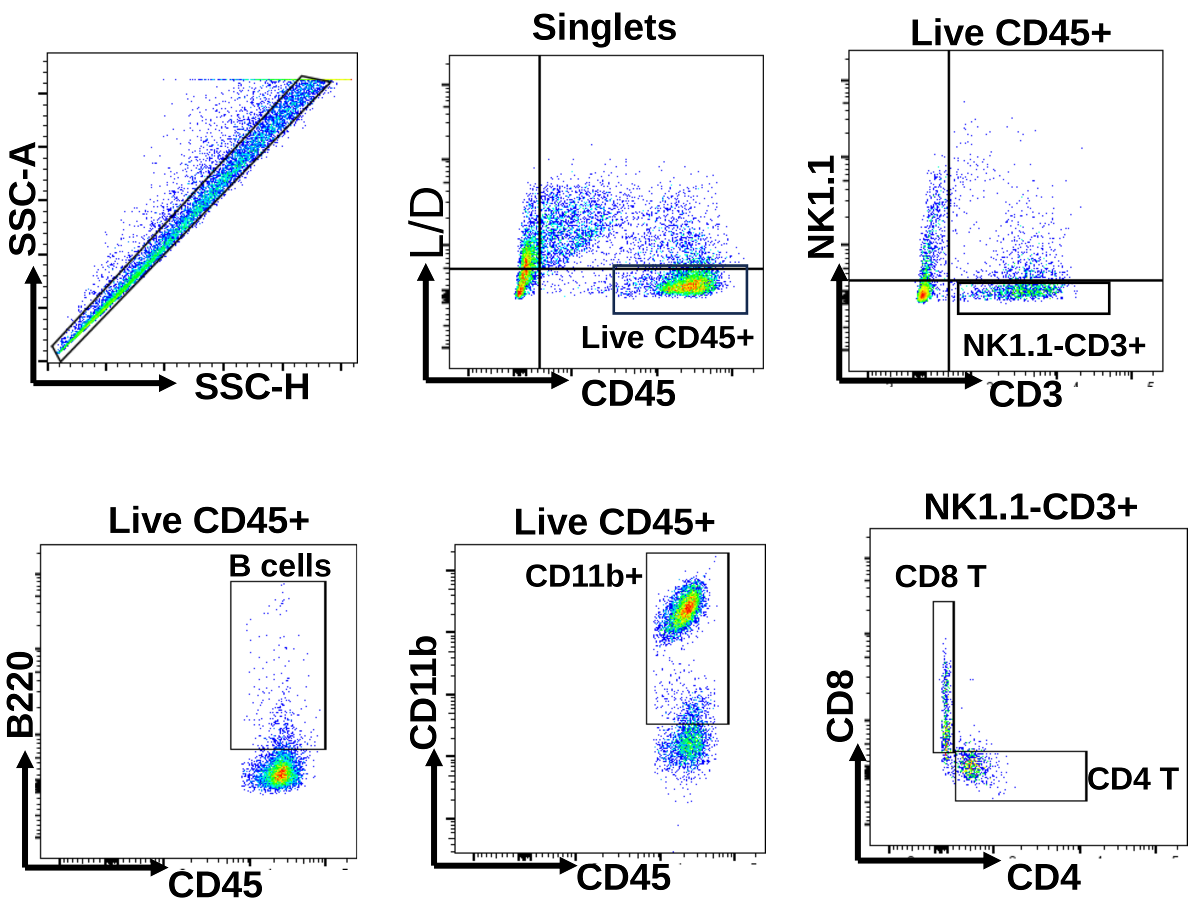

Fig. S1. Tumor Immune cells gating strategy. Tumor single cells stained from 2-week time point to study overall immune lineage such as B cells (B220+), CD4 and CD8 T cells, Myeloid cells (CD11b+) which are singlets and Immune cell marker positive (CD45+) live cells.

bioRxiv preprint doi: https://doi.org/10.1101/2024.05.03.592354; this version posted May 5, 2024. The copyright holder for this preprint (which was not certified by peer review) is the author/funder, who has granted bioRxiv a license to display the preprint in perpetuity. It is made available under aCC-BY 4.0 International license.

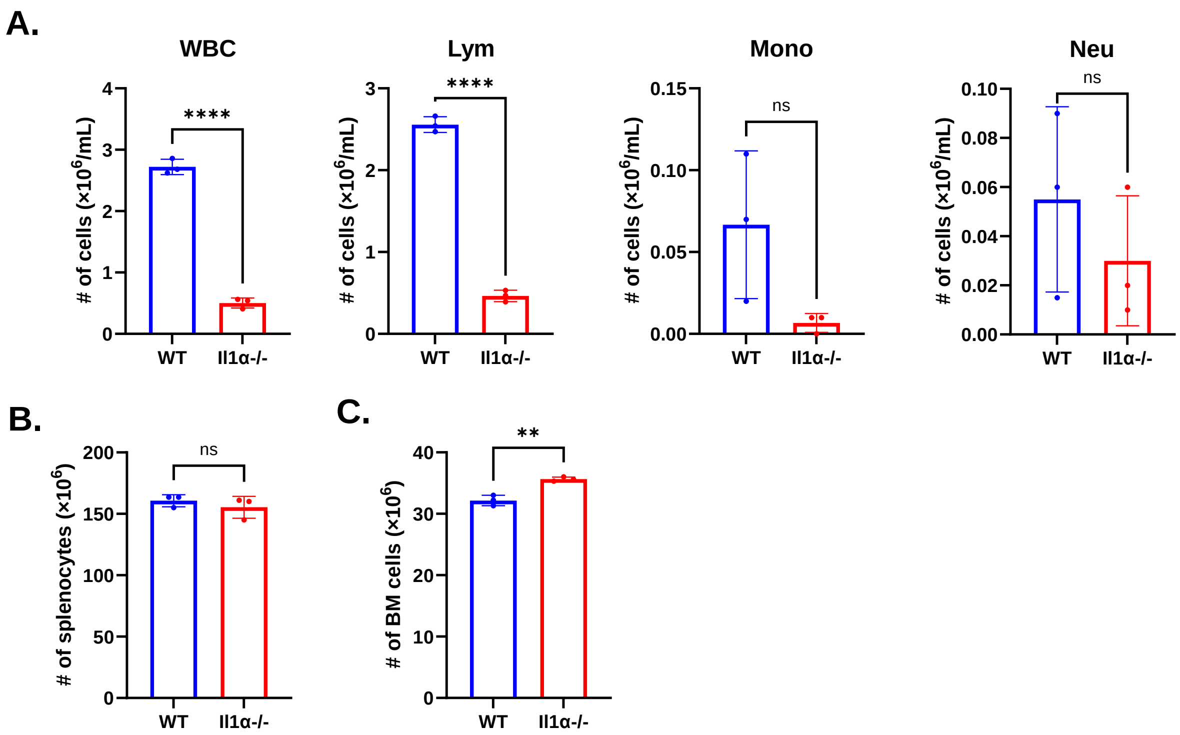

Fig. S2. Immune profiles of tumor-free WT and Il1α$^{-/-}$ mice. A. Blood Vetscan® data shown between tumor-free WT and *Il1α*$^{-/-}$ mice. **B.** and **C** describe the total number of spleen and bone marrow leukocytes from tumor-bearing WT and *Il1α*$^{-/-}$ mice. Unpaired Two Tailed t-test was performed. **** p<0.0001; **p < 0.01; ns, not significant.

bioRxiv preprint doi: https://doi.org/10.1101/2024.05.03.592354; this version posted May 5, 2024. The copyright holder for this preprint (which was not certified by peer review) is the author/funder, who has granted bioRxiv a license to display the preprint in perpetuity. It is made available under aCC-BY 4.0 International license.

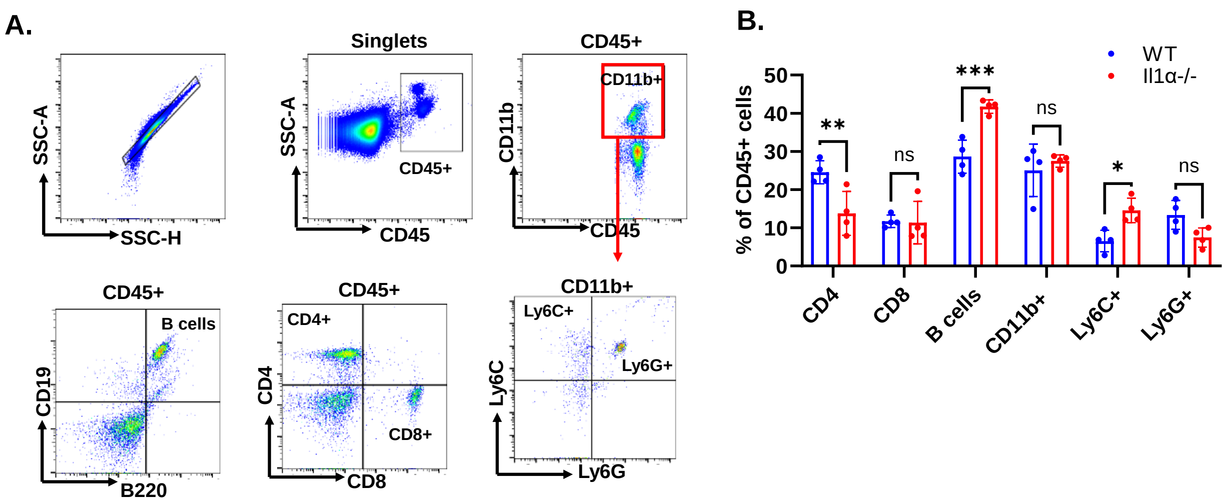

Fig. S3. Blood Immune cells gating strategy. A. Blood collected from 2-week time point to study overall immune lineage such as B cells (B200+), CD4 and CD8 T cells, Myeloid cells (CD11b+) and their subsets Ly6C+ and Ly6G+ which are singlets and Immune cell marker positive (CD45+) live cells **B**. Quantified distribution of immune cells in blood comparing *WT* and *Il1α*$^{-/-}$. Unpaired Two Tailed t-test was performed. ***<0.001; **<0.01.

bioRxiv preprint doi: https://doi.org/10.1101/2024.05.03.592354; this version posted May 5, 2024. The copyright holder for this preprint (which was not certified by peer review) is the author/funder, who has granted bioRxiv a license to display the preprint in perpetuity. It is made available under aCC-BY 4.0 International license.

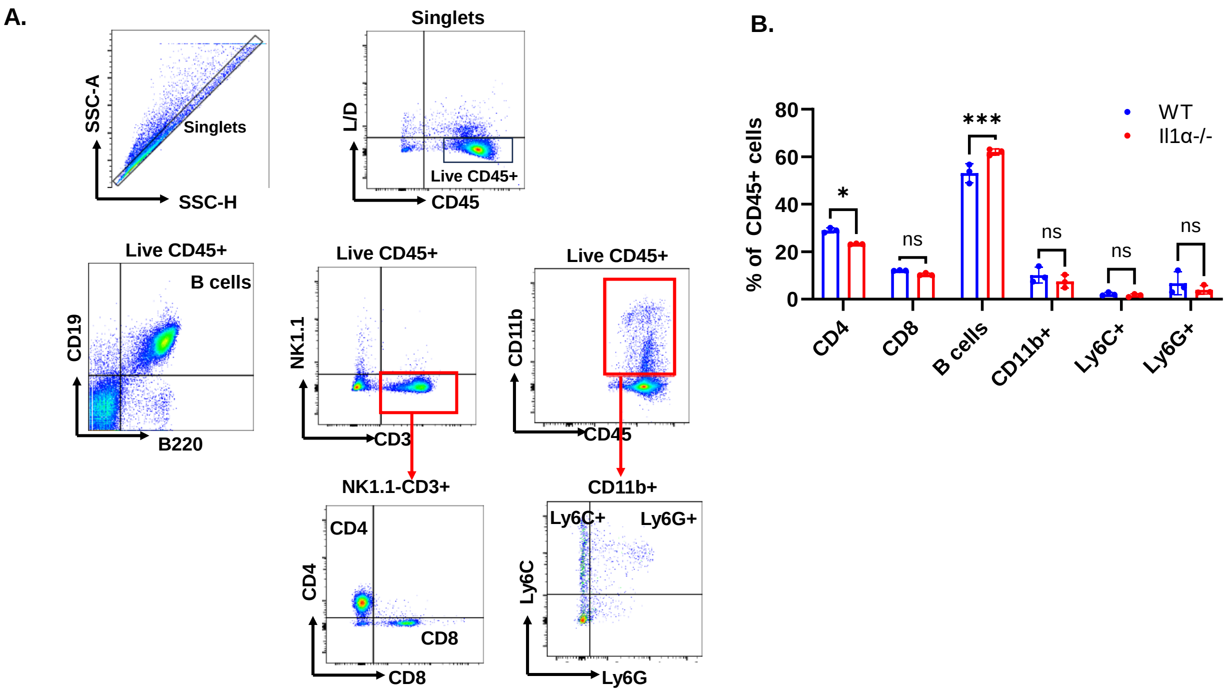

Fig. S4. Spleen Immune cells gating strategy. A. Spleen from 2-week time point to study overall immune lineage such as B cells (B200+), CD4 and CD8 T cells, Myeloid cells (CD11b+) and their subsets Ly6C+ and Ly6G+ which are singlets and Immune cell marker positive (CD45+) live cells **B**. Quantified distribution of immune cells in spleen comparing *WT* and *Il1α-/-*. Unpaired Two Tailed t-test was performed. ***<0.001; **<0.01.

bioRxiv preprint doi: https://doi.org/10.1101/2024.05.03.592354; this version posted May 5, 2024. The copyright holder for this preprint (which was not certified by peer review) is the author/funder, who has granted bioRxiv a license to display the preprint in perpetuity. It is made available under aCC-BY 4.0 International license.

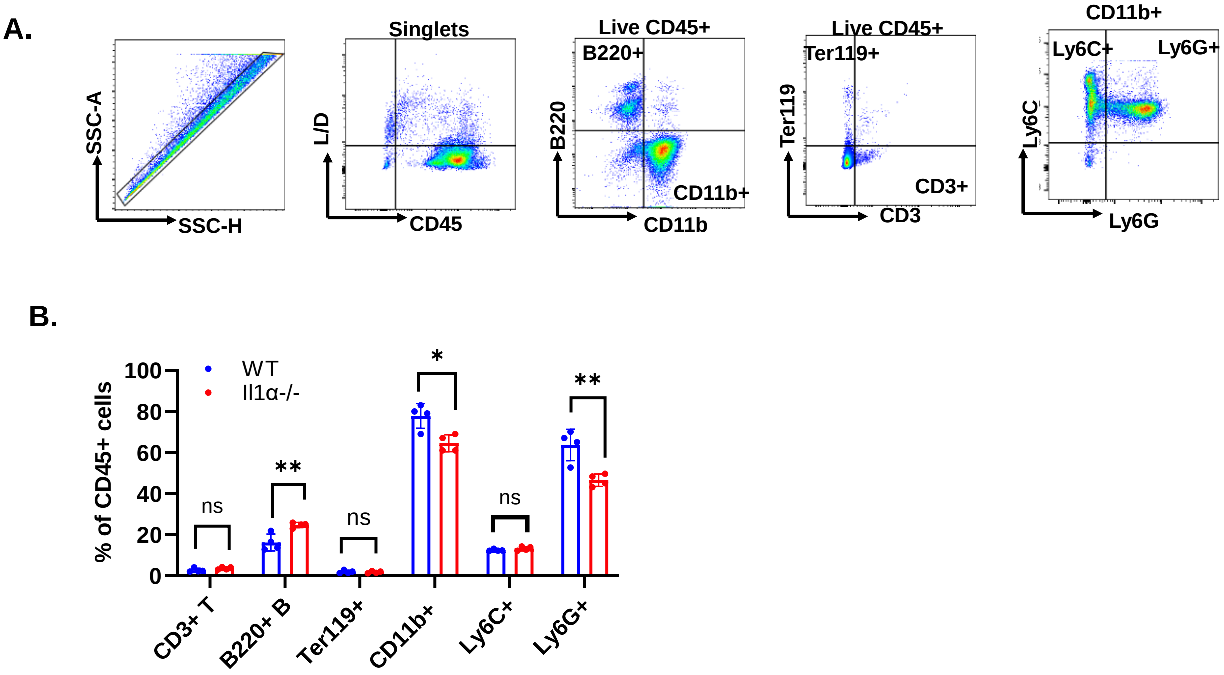

Fig. S5. Bone Marrow Immune cells gating strategy. A. Bone marrow from 2-week time point to study overall immune lineage such as B cells (B200+), CD3+ T cells, Myeloid cells (CD11b+), Erythrocytes (Ter119+) and subsets of myeloid cells Ly6C+ and Ly6G+ which are singlets and Immune cell marker positive (CD45+) live cells. **B**. Quantified distribution of immune cells in bone marrow comparing *WT* and *Il1α*$^{-/-}$ .Unpaired Two Tailed t-test was performed. ***<0.001; **<0.01.

bioRxiv preprint doi: https://doi.org/10.1101/2024.05.03.592354; this version posted May 5, 2024. The copyright holder for this preprint (which was not certified by peer review) is the author/funder, who has granted bioRxiv a license to display the preprint in perpetuity. It is made available under aCC-BY 4.0 International license.

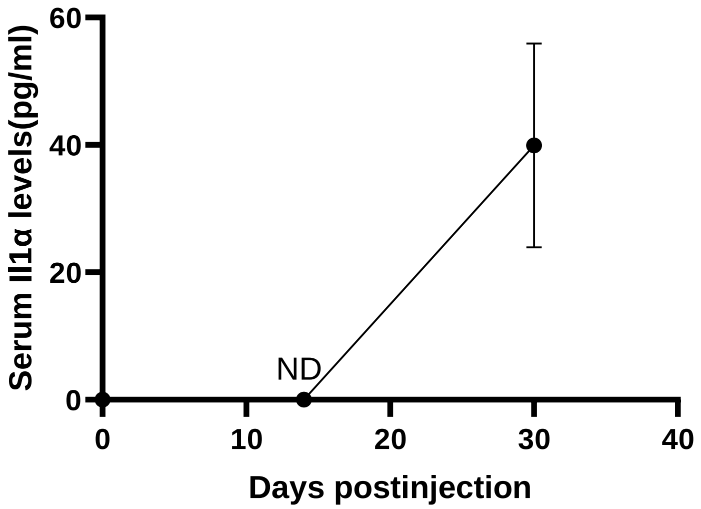

Fig. S6. Il1α plasma dynamics. Plasma was monitored from day 0 until day 30 from *WT* tumor bearing mice quantified using Multiplex ELISA assay for Il1α (n = 3). ND, not detectable using this technology. The expression levels are not notably elevated compared to the background.

bioRxiv preprint doi: https://doi.org/10.1101/2024.05.03.592354; this version posted May 5, 2024. The copyright holder for this preprint (which was not certified by peer review) is the author/funder, who has granted bioRxiv a license to display the preprint in perpetuity. It is made available under aCC-BY 4.0 International license.

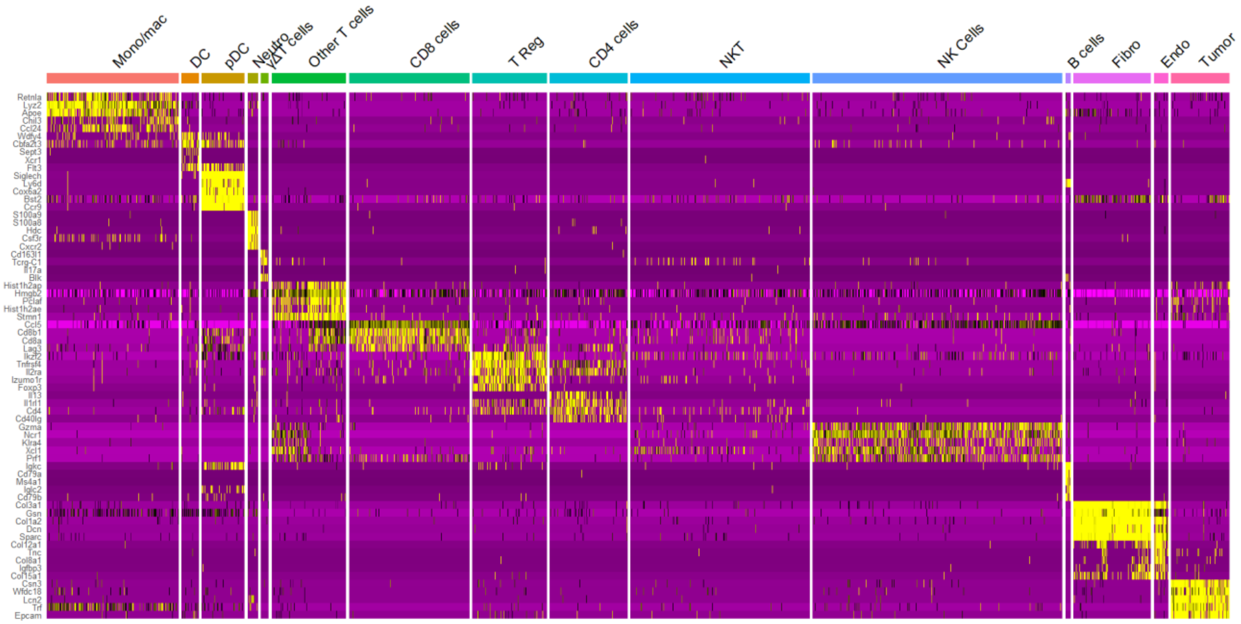

Fig. S7. The top unique genes from the 14 overall clusters shown in heat map from scRNA seq analysis performed on 2-week tumor.

bioRxiv preprint doi: https://doi.org/10.1101/2024.05.03.592354; this version posted May 5, 2024. The copyright holder for this preprint (which was not certified by peer review) is the author/funder, who has granted bioRxiv a license to display the preprint in perpetuity. It is made available under aCC-BY 4.0 International license.

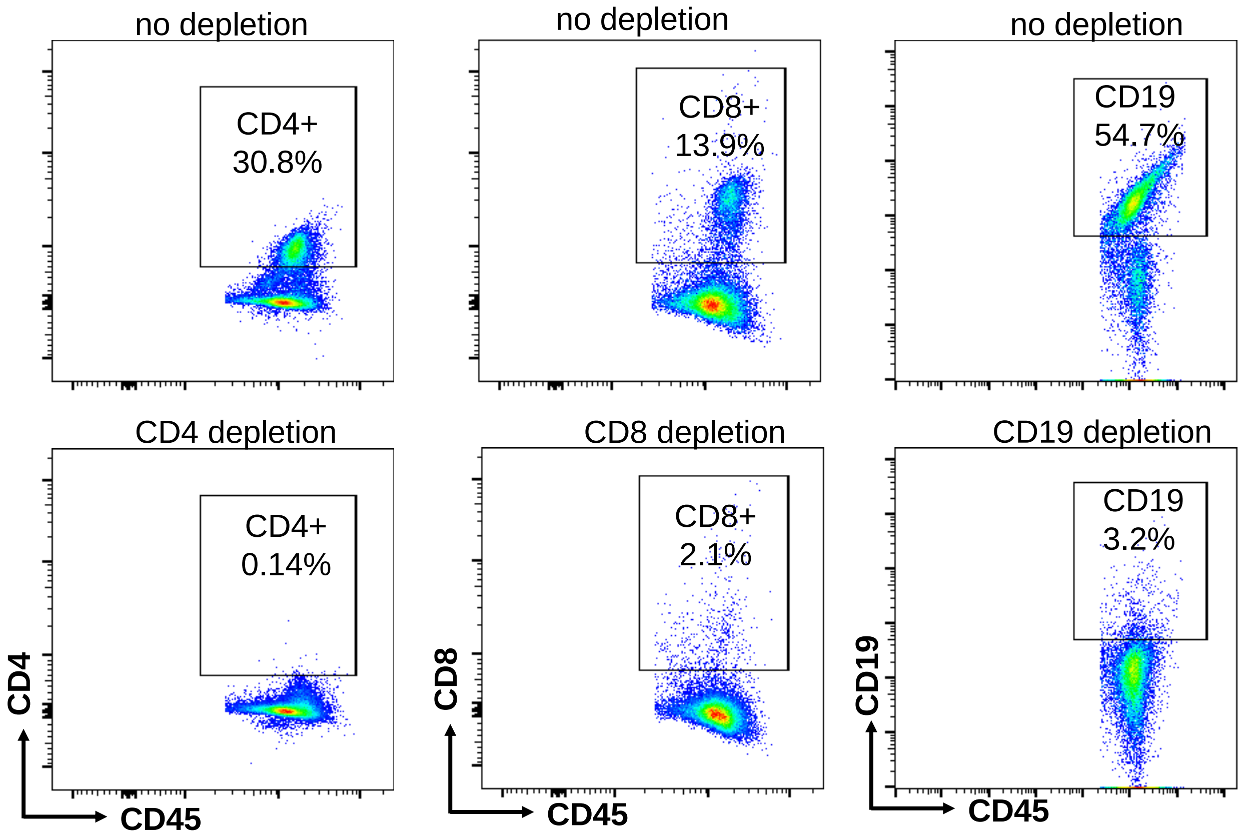

Fig. S8. Immune cell depletion validation. Representative flow cytometry data of spleen to indicate depletion of CD4, CD8 and CD19 using neutralization antibody pre-gated for CD45+ live cells.

bioRxiv preprint doi: https://doi.org/10.1101/2024.05.03.592354; this version posted May 5, 2024. The copyright holder for this preprint (which was not certified by peer review) is the author/funder, who has granted bioRxiv a license to display the preprint in perpetuity. It is made available under aCC-BY 4.0 International license.

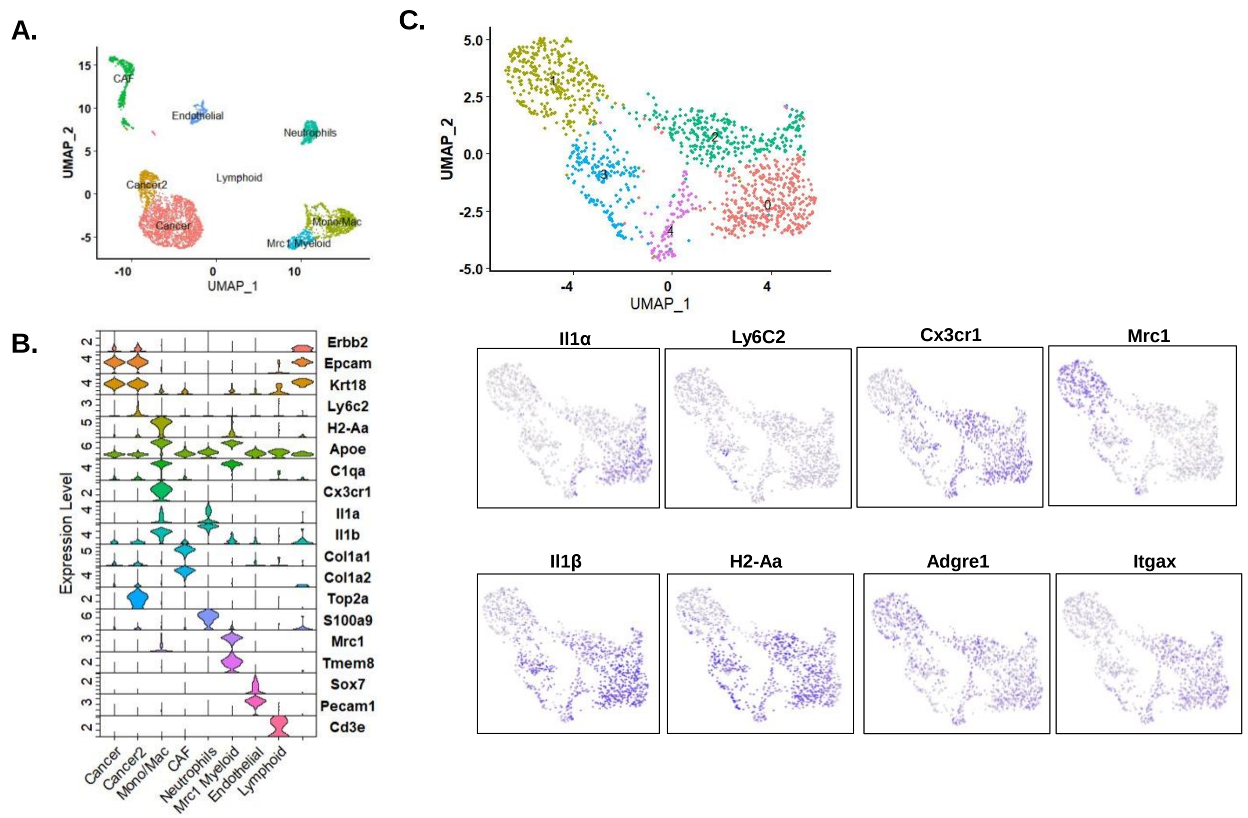

Fig. S9. Cross verified MMTV driven HER2+ mice tumor scRNA seq dataset for Il1α source from GEO accession number: GSE166321. **A.** The representative tSNE, **B.** The top genes are shown in violin plot to identify the clusters. **C.** The Mono/Mac and Mrc1+ myeloid clusters were reclustered to find 5 subclusters as shown in the UMAP with genes Il1a, Ly6c2, Cx3cr1, Mrc1, Il1b, H2-Aa, Adgre1 and Itgax.

bioRxiv preprint doi: https://doi.org/10.1101/2024.05.03.592354; this version posted May 5, 2024. The copyright holder for this preprint (which was not certified by peer review) is the author/funder, who has granted bioRxiv a license to display the preprint in perpetuity. It is made available under aCC-BY 4.0 International license.

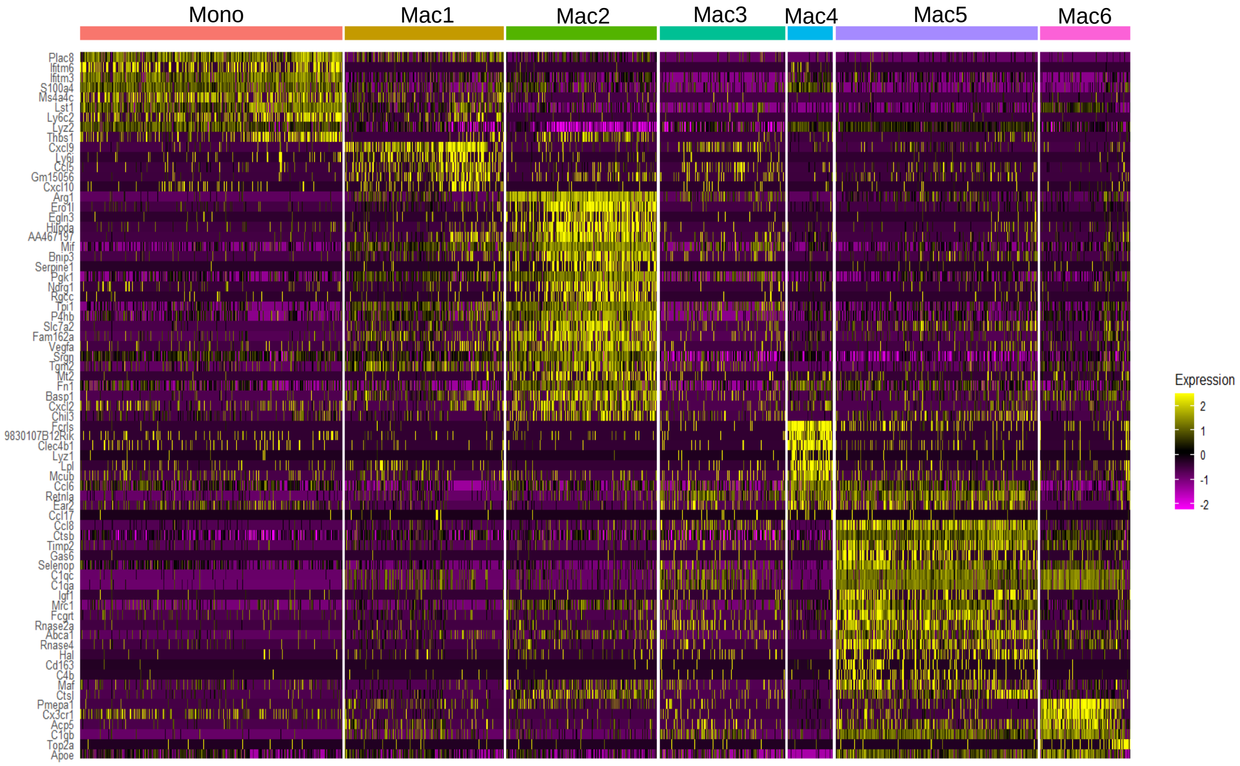

Fig. S10. Top unique genes from the reclustered mono/mac subclusters from the 2-week time point tumor shown as heat map using scRNA seq analysis.

bioRxiv preprint doi: https://doi.org/10.1101/2024.05.03.592354; this version posted May 5, 2024. The copyright holder for this preprint (which was not certified by peer review) is the author/funder, who has granted bioRxiv a license to display the preprint in perpetuity. It is made available under aCC-BY 4.0 International license.

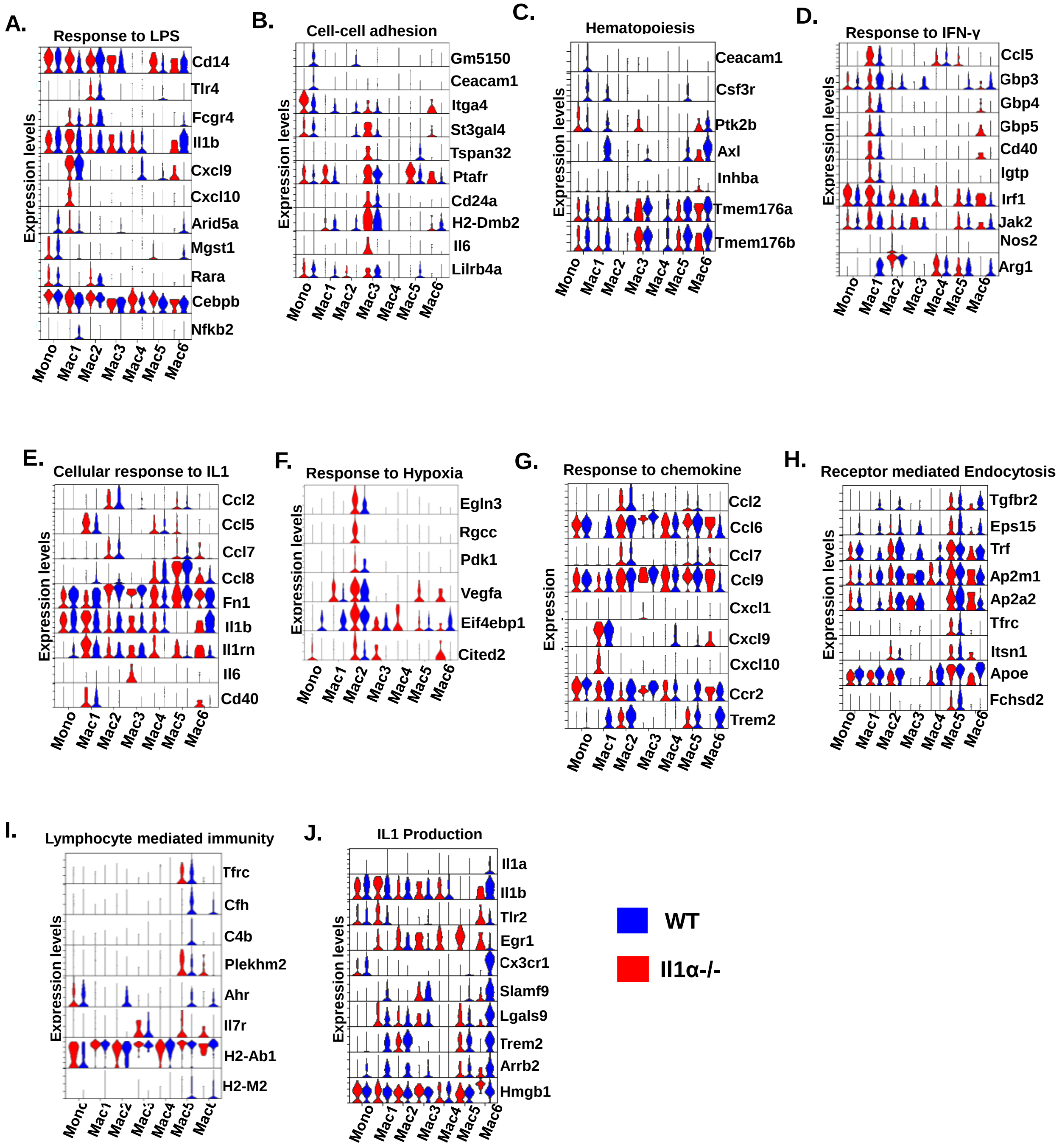

Fig. S11. Unique genes selected to show from GO-pathway analysis with respect to WT (Blue) and *Il1α*$^{-/-}$ (Red) in Violin plot from scRNA seq analysis.

bioRxiv preprint doi: https://doi.org/10.1101/2024.05.03.592354; this version posted May 5, 2024. The copyright holder for this preprint (which was not certified by peer review) is the author/funder, who has granted bioRxiv a license to display the preprint in perpetuity. It is made available under aCC-BY 4.0 International license.

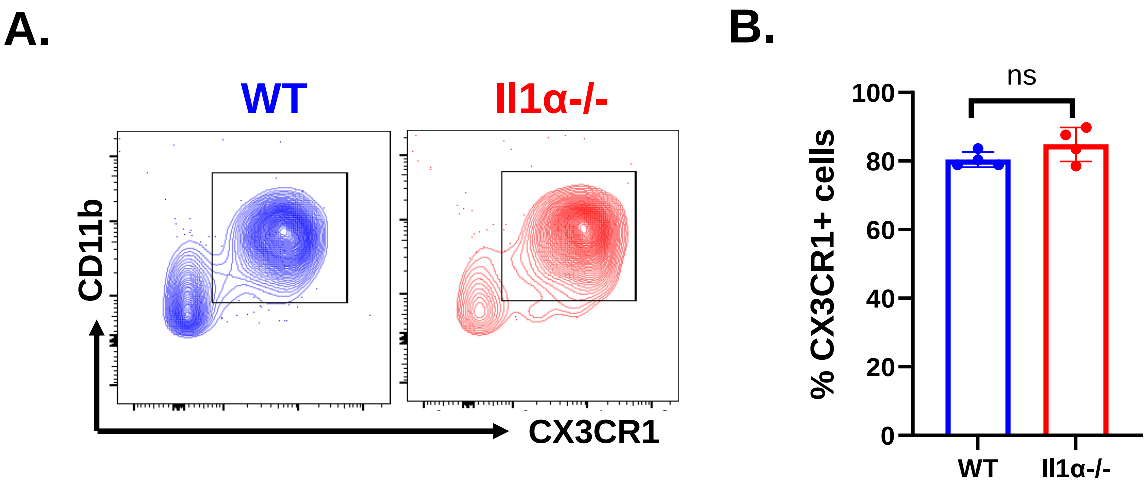

Fig. S12.CX3CR1+ myeloid cells in blood from 2-week time point are not quantitively different between the groups A. Representative figure of CD11b+Cx3cr1+ flow cytometry from blood which is pre-gated from CD45+ live cells. **B.** Quantified percent of CD11b+Cx3cr1+ cells in blood n = 4. Unpaired Two Tailed t-test was performed.

bioRxiv preprint doi: https://doi.org/10.1101/2024.05.03.592354; this version posted May 5, 2024. The copyright holder for this preprint (which was not certified by peer review) is the author/funder, who has granted bioRxiv a license to display the preprint in perpetuity. It is made available under aCC-BY 4.0 International license.

Table S1. Abbreviations for stimuli used for induction of human macrophage polarization.

MCSF	macrophage colony-stimulating factor
GMCSF	granulocyte-macrophage colony-stimulating factor
GC	glucocorticoid
IC	immune complexes
P3C	Pam3CysSerLys4
TPP	TNF+PGE2+P3C
PA	palmitic aicd
OA	oleic acid
LA	lauric acid
LiA	linoleic acid
SA	stearic acid
sLPS	standard lipopolysaccharide
upLPS	ultrapure lipopolysaccharide
HDL	high density lipoprotein
MCD	cyclodextrins

bioRxiv preprint doi: https://doi.org/10.1101/2024.05.03.592354; this version posted May 5, 2024. The copyright holder for this preprint (which was not certified by peer review) is the author/funder, who has granted bioRxiv a license to display the preprint in perpetuity. It is made available under aCC-BY 4.0 International license.

Table S2. List of antibodies used in the study.

**Target**	**Host**	**Clone**	**Flour**	**Catalog Number**	**Company**	**Final Dilution**
Arg1	Rat	A1exF5	PE	12-3697-80	Invitrogen	1:100
B220	Rat	RA3-6B2	FITC	103206	Biolegend	1:200
CD11b	Rat	M1/70	FITC	101206	Biolegend	1:400
CD11b	Rat	M1/70	APC	101212	Biolegend	1:400
CD11b	Rat	M1/70	PE	101208	Biolegend	1:400
CD11c	Armenian Hamster	N418	PE/Cy7	117318	Biolegend	1:100
CD11c	Armenian Hamster	N418	BV421	117330	Biolegend	1:100
CD19	Rat	6D5	PE/Cy7	115520	Biolegend	1:100
CD3	Armenian Hamster	145-2C11	PE	100308	Biolegend	1:400
CD3	Rat	17A2	FITC	100204	Biolegend	1:400
CD4	Rat	RM4-5	FITC	100510	Biolegend	1:100
CD40L	Rat	SA047C3	PE/Cy7	157008	Biolegend	1:100
CD44	Rat	IM7	BV510	103044	Biolegend	1:100
CD45	Rat	30-F11	APC	103112	Biolegend	1:400
CD8a	Rat	53-6.7	APC/Cy7	100714	Biolegend	1:100
CTLA4/ CD152	Armenian Hamster	UC10-4B9	BV421	106312	Biolegend	1:100
CX3CR1	Mouse	SA011F11	BV421	149023	Biolegend	1:100
CX3CR1	Mouse	SA011F11	PE/Cy7	149016	Biolegend	1:100
F4/80	Rat	BM8	APC/Cy7	123118	Biolegend	1:100
F4/80	Rat	BM8	BV510	123135	Biolegend	1:100
F4/80	Rat	BM8	PE	123110	Biolegend	1:100
GR1	Rat	RB6-8C5	PE	108408	Biolegend	1:400
Granzyme B	Mouse	OA16A02	PE/Cy7	372214	Biolegend	1:100
IFNγ	Rat	XMG1.2	BV421	505830	Biolegend	1:100
IL1a	Armenian Hamster	ALF-161	PE	503203	Biolegend	1:100
IL1b	Rat	NJTEN3	PE	12-7114-80	Invitrogen	1:100

bioRxiv preprint doi: https://doi.org/10.1101/2024.05.03.592354; this version posted May 5, 2024. The copyright holder for this preprint (which was not certified by peer review) is the author/funder, who has granted bioRxiv a license to display the preprint in perpetuity. It is made available under aCC-BY 4.0 International license.

iNOS	Rat	W16030C	PE	696806	Biolegend	1:100
KI67	Rat	16A8	BV421	652411	Biolegend	1:100
Ly6C	Rat	HK1.4	BV785	128041	Biolegend	1:100
Ly6C	Rat	HK1.4	PE/Cy7	128018	Biolegend	1:100
LY6G	Rat	1A8	APC/Cy7	127624	Biolegend	1:100
LY6G	Rat	1A8	BV510	127633	Biolegend	1:100
LY6G	Rat	1A8	BUV387	127678	Biolegend	1:100
MHCII	Rat	M5/114.15.2	PE/Cy7	107630	Biolegend	1:200
MHCII	Rat	M5/114.15.2	FITC	107606	Biolegend	1:200
NK1.1	Mouse	S17016D	PE/Cy7	156514	Biolegend	1:100
PD-1	Rat	RMP1-30	PerCP/Cy5.5	109120	Biolegend	1:100
Ter119	Rat	TER-119	PE/Cy7	116221	Biolegend	1:100
CD4	Rat		GK1.5	BE0003-1	Bioxcell	
CD8a	Rat		2.43	BE0061	Bioxcell	
CD19	Mouse		4G7	BE0281	Bioxcell	

bioRxiv preprint doi: https://doi.org/10.1101/2024.05.03.592354; this version posted May 5, 2024. The copyright holder for this preprint (which was not certified by peer review) is the author/funder, who has granted bioRxiv a license to display the preprint in perpetuity. It is made available under aCC-BY 4.0 International license.

Table S3. List of key reagents used in this study.

**Reagent**	**Supplier**	**Catalog Number**	**Final Dilution**
DMEM/F12	Corning	10-090-CV	
RPMI	Corning	10-040-CV	
Fetal Bovine Serum	Peak Bio	PS-FB3	
Recombinant Human Insulin	Gibco	Rp-10908	
Phosphate Buffered Saline	Corning	21040CV	
Penicillin/Steptomycin Solution	Corning	30-002-CI	
Antibiotic Antimycotic Solution	Corning	30-004-CI	
0.25% Trypsin	Gibco	25200-072	
Matrigel Matrix	Corning	CB-40234	
1ml 25G syringe	BD	309626	
0.5ml 28G syringe	BD	329461	
EDTA	VWR	E522-100ML	
PierceTM Protein Inhibitor tablets	Thermo Scientific	A32955	
EDTA Blood Collection Tubes	BD	BD367842	
Vetscan HM5 Hematology Analyzer	Abaxis	770-0000	
10X RBC lysis buffer	Biolegend	420302	
4% Paraformaldehyde in PBS	Thermo Scientific	AAJ61899AK	
No.9 Razor Blades	Garvey	40475	
5cm culture dish	Corning	430166	
15ml tubes	Extra Gene	P1013-15BF	
Collagenase IV	Worthington Biochem	LS004188	
Hyaluronidase	MP Biochemicals	100740	
40Micron Filter	VWR	21008-949	
Sucrose	Macron	MK772304	
Peel-A-Way OCT Mold	Polysciences	18646A	
Tissue-Tek OCT compound	Sakura	4583	
Cryosection station	Leica	CM1860UV	
ColorMark Charged Glass Slides	Epredia	CM-4951WPLUS-001	
Uncharged Glass Slides	Fishcer Scientific	12-544-4	
Cell Counter	Bio-Rad	TC20	
Flow cytometry Machine	BD	FACSAria II	
Flow cytometry Machine	BD	Symphony A5	
Cell sorter	Sony	SH800	
Zombie Aqua	Invitrogen	L34957	1:1000

bioRxiv preprint doi: https://doi.org/10.1101/2024.05.03.592354; this version posted May 5, 2024. The copyright holder for this preprint (which was not certified by peer review) is the author/funder, who has granted bioRxiv a license to display the preprint in perpetuity. It is made available under aCC-BY 4.0 International license.

Zombie Yellow	Invitrogen	L34968	1:1000
7-AAD	Biolegend	420404	1:1000
Cytofix/cytoperm plus kit	BD	555028	
Foxp3 intracellular stain kit	eBioscience	00-5523-00	
cd16/32 receptor blockade	BD	553142	1:1000
CD8 isolation kit	Miltenyi Biotec	130-104-075	
carboxyfluorescein diacetate succinimidyl ester (CFSE)	eBioscience	65-0850-84	
Anti-CD3/CD28 beads	Miltenyi Biotec	130-093-627	
